# LncRNAs has been identified as regulators of Myeloid-derived suppressor cells in lung cancer

**DOI:** 10.3389/fimmu.2023.1067520

**Published:** 2023-02-02

**Authors:** Yifan Liu, Yukun Han, Yanhua Zhang, Tongtong Lv, Xiaochun Peng, Jinbai Huang

**Affiliations:** ^1^ Department of Oncology, Jingzhou Hospital Affiliated to Yangtze University, Jingzhou, Hubei, China; ^2^ Department of Pathophysiology, School of Basic Medicine, Health Science Center, Yangtze University, Jingzhou, Hubei, China; ^3^ Department of Medical Imaging, School of Medicine, and Positron Emission Computed Tomography (PET) Center of the First Affiliated Hospital, Yangtze University, Jingzhou, Hubei, China; ^4^ Laboratory of Oncology, Center for Molecular Medicine, School of Basic Medicine, Health Science Center, Yangtze University, Jingzhou, Hubei, China

**Keywords:** lncRNA, MDSC, lung cancer, targeted therapy, immunotherapy

## Abstract

Lung tumours are widespread pathological conditions that attract much attention due to their high incidence of death. The immune system contributes to the progression of these diseases, especially non-small cell lung cancer, resulting in the fast evolution of immune-targeted therapy. Myeloid-derived suppressor cells (MDSCs) have been suggested to promote the progression of cancer in the lungs by suppressing the immune response through various mechanisms. Herein, we summarized the clinical studies on lung cancer related to MDSCs. However, it is noteworthy to mention the discovery of long non-coding RNAs (lncRNAs) that had different phenotypes and could regulate MDSCs in lung cancer. Therefore, by reviewing the different phenotypes of lncRNAs and their regulation on MDSCs, we summarized the lncRNAs’ impact on the progression of lung tumours. Data highlight LncRNAs as anti-cancer agents. Hence, we aim to discuss their possibilities to inhibit tumour growth and trigger the development of immunosuppressive factors such as MDSCs in lung cancer through the regulation of lncRNAs. The ultimate purpose is to propose novel and efficient therapy methods for curing patients with lung tumours.

## 1 Introduction

Concerning cancer deaths, lung tumours are recognized as one of the leading causes globally. They are further classified in the category as conditions whose rates of morbidity and mortality, as well as the degrees of malignancy, are the highest. Along with the industrialization progress and environmental changes, the aetiology of lung cancer has become even more complex (1). According to sources, in 2018, many patients diagnosed with non-small cell lung cancer (NSCLC) in stage III were recorded, corresponding to 80% of all cases (2). Despite the ongoing efforts to develop efficient anti-cancer therapies, NSCLC has become among the most lethal cancers worldwide (3). In recent years, tumour-related immunosuppression has become the focal point in targeted therapy for lung tumours. In this respect, bone marrow-derived suppressor cells (MDSCs) have attracted much attention. As early as 1970, studies have pointed out the MDSCs relation to tumour development (4). Its role in the tumour microenvironment has been pivotal and turned into a new target for cancer therapy (5). In a study by Gabrilovich and Nagaraj, the authors confirmed that MDSCs are heterogeneous cell populations derivative from the bone marrow precursor and immature cells (IMC). Under standard conditions, IMC can quickly specialize into dendritic cells (DC), mature granulocytes, and macrophages. On the occasions of cancer, infection, inflammation or other illnesses, the authors have reported an increase in the number of MDSCs and inhibition of IMC differentiation into developed cells of the bone marrow (6).

Moreover, MDSCs are known for their potent immunosuppressive function. Immunotherapy with immune checkpoint inhibitors has been reported to control the long-term effects of tumours to a certain extent. However, due to the potential repercussions of MDSCs’ massive expansion, the MDSCs-induced immunosuppression has been considered the mechanism that effectively hinders the immune checkpoint blockade (7) Regarding lncRNAs, they have been implicated in the progression of tumours and various roles depending on the different expression types. Importantly, lncRNAs have multiple functions that are not limited to regulating the MDSCs generation, recruitment and immunosuppression. They also target multiple pathways simultaneously. The latter allows lncRNAs to act as markers in diagnosing tumours and are highly valued for targeted therapy (8). The modulatory effect of lncRNAs on MDSCs and the regulation of lung tumour tissue may open new horizons in treating individuals with lung tumours. The current paper is mainly based on the targeted therapy of MDSCs that lncRNAs have regulated to change the survival rate of patients with lung tumours. First, to elaborate on the importance of MDSCs in patients diagnosed with lung tumours, we have analyzed the MDSCs mechanism and investigated the clinical studies focused on the matter. Secondly, we studied the role of differential lncRNA expression in these cells and the impact on tumour progression. Finally, we have included clinical studies in which lncRNAs regulated various tumours, justifying their potential therapeutic value in tumours.

## 2 The roles of myeloid-derived suppressor cells (MDSCs) in lung tumours

### 2.1 Phenotype of MDSCs

Cell heterogeneity is evident in MDSCs as they include two major subpopulations, those of granulocytes (G-MDSCs) and monocytes (M-MDSCs) in both human and animal (mice) models. They are derived from granulocytes or monocytes and represent relatively stable forms of pathologic activation of these blood cell populations (9). In mice, CD11b^+^ Ly6G-Ly6C^high^ and CD11b^+^Ly6G^+^Ly6C^low^ stand for the phenotype of M-MDSCs and G-MDSCs, respectively. Related studies have confirmed CD11b^+^ GR-1^low^cells’ ability as the most efficient in suppressing the immune system in contrast to CD11b^+^ GR-1^high^ cells, regarded as the least effective. In humans, the MDSCs’ complexity is even greater and constitutes CD11b^+^CD14^+^HLA-DR^low/neg^ M-MDSCs and CD11b^+^CD14^-^CD15^+^ G-MDSCs populations. The MDSCs in cancer patients express granulocyte markers and bone marrow cell markers like CD11b and CD33, which are the most common. However, there is still a need for a profound exploration of the surface markers of M-MDSCs and G-MDSCs due to the differentiation of the MDSCs’ phenotype in various diseases (10, 11).Besides, a small group of bone marrow progenitors with MDSCs characteristics is identified only in humans and is named the “early MDSC” group. The group is mainly composed of bone marrow progenitors and precursors that account for less than 5% of the total number of MDSCs (12). However, many ongoing efforts have been made to report the surface markers of MDSCs. With fluorescence-activated cell sorting (FACS) for evaluating the multicolour immunofluorescence staining, several phenotypes of MDSCs in lung cancer have been reported (13).

### 2.2 Mechanism of action of MDSCs

The potent immunosuppressive effects of MDSCs, are present in most cancers (14). For example, the tumour microenvironment (TME) comprises different cell populations in a complex matrix. Cellular components of TME are markers that can regulate cancer processes like tumour proliferation, angiogenesis, invasion and metastasis, chemotherapy resistance, etc. Therefore, TME has become a new target for cancer treatment (15, 16) Furthermore, during tumour progression, the cells undergo alternations in their metabolism to satisfy their energy needs, with the ultimate goal of achieving proliferation and differentiation of the tumour—the last results in nutrition competition between the immune cells and immune modulators in the TME of MDSCs. Thus, increased glycolysis, fatty acid metabolism, and up-regulation of enzymes that are essential metabolites of catabolism are observed in the MDSCs tumour microenvironment. The last grants MDSCs immunosuppressive function (17). Other investigations have demonstrated that the MDSCs’ immunomodulatory role is mainly based on the inhibition of T cells (18).This happens by various mechanisms. The first one is described by the production of reactive oxygen species (ROS), and reactive nitrogen species (RNS) can result in blocking T cells activation and function by MDSCs. As reported by Wang et al., G-MDSCs are mainly responsible for producing ROS and arginase-1 (ARG-1).

In contrast, M-MDSCs mainly produce ARG-1 and nitric oxide (NO) to exert immunosuppressive effects (19). The half-life of the produced NO is more extended, while it requires cell entry. Nonetheless, there is no requirement for close contact between M-MDSCs and T cells, enabling M-MDSCs effectively inhibit non-specific T cell responses (20). One prominent feature of MDSCs is the up-regulation of ROS produced by G-MDSCs in mice and individuals diagnosed with cancer. The immunosuppressive effect of MDSCs can be significantly enhanced by the expression of ROS in cancer patients and mice (21). Concretely, superoxide anion (O2^-^), hydrogen peroxide (H_2_O_2_) and peroxynitrite (PNT) represent the abovementioned family of ROS. However, NO reacts with O2^-^ to form PNT, which then prevents the recognition of the antigen/major histocompatibility complex (MHC) peptide by nitrification of the MHC class I molecules and the T cell receptors (TCRs). They reduce the TCR affinity for antigen-MHC complex and block T cell migration by nitrification of T cell-specific chemokines (22, 23). The second mechanism providing MDSCs with immunomodulatory roles includes the depletion of L-cysteine and arginine by MDSCs, which are required for the proliferation and activation of T cell (24). The ARG-1 produced by G-MDSCs and M-MDSCs metabolizes L-arginine to L-ornithine in the urea cycle, making L-arginine induce its exhaustion in the TME. Other studies have revealed that PEGylated forms of Arg I (PEG-ARG I) in mice promote tumour growth. This is accompanied by augmented amounts of MDSCs (25).On the other hand, cysteine’s role in T cell activation is indispensable. Factually, macrophages and DCs deliver to T cells the obtained cysteine, which the DCs and macrophages metabolise to methionine during the standard antigen processing and presentation. Moreover, in TME is observed a reduction in the activation of T cells and cysteine production by DCs as a result of a deprivation of macrophages of cysteine and DCs. The above is due to a large number of cysteine depletion by MDSCs (26).

The third mechanism includes the interaction between MDSCs with T cells that migrate to the lymph glands and T-cell activity *via* the expression of ADAM17 (metalloprotease structural domain 17 and a disintegrin) on their cell membrane. The MDSCs interfere. It has been reported that the latter leads to the downregulation of the homing receptor CD62L (L-selectin) on T cells (24).When it comes to effective anti-cancer immunity, and for purposes of action, it requires the transportation to the tumour of activated T cells and the activation of tumour-reactive T cells. Meanwhile, CD62L has been regarded as an essential molecule in this process, directing primitive lymphocytes to lymph glands in the periphery.

Furthermore, CD62L has proven necessary for housing the primitive T cells in the lymph glands, activating molecules on and in the cell membrane. Conversely, the suppressed activity of CD62L on primitive T cells by MDSCs decreases the chance of primitive T cells being delivered to the activation site. The last functions as MDSCs’ suppressive anti-tumour immune response (27).

**Figure 1:**
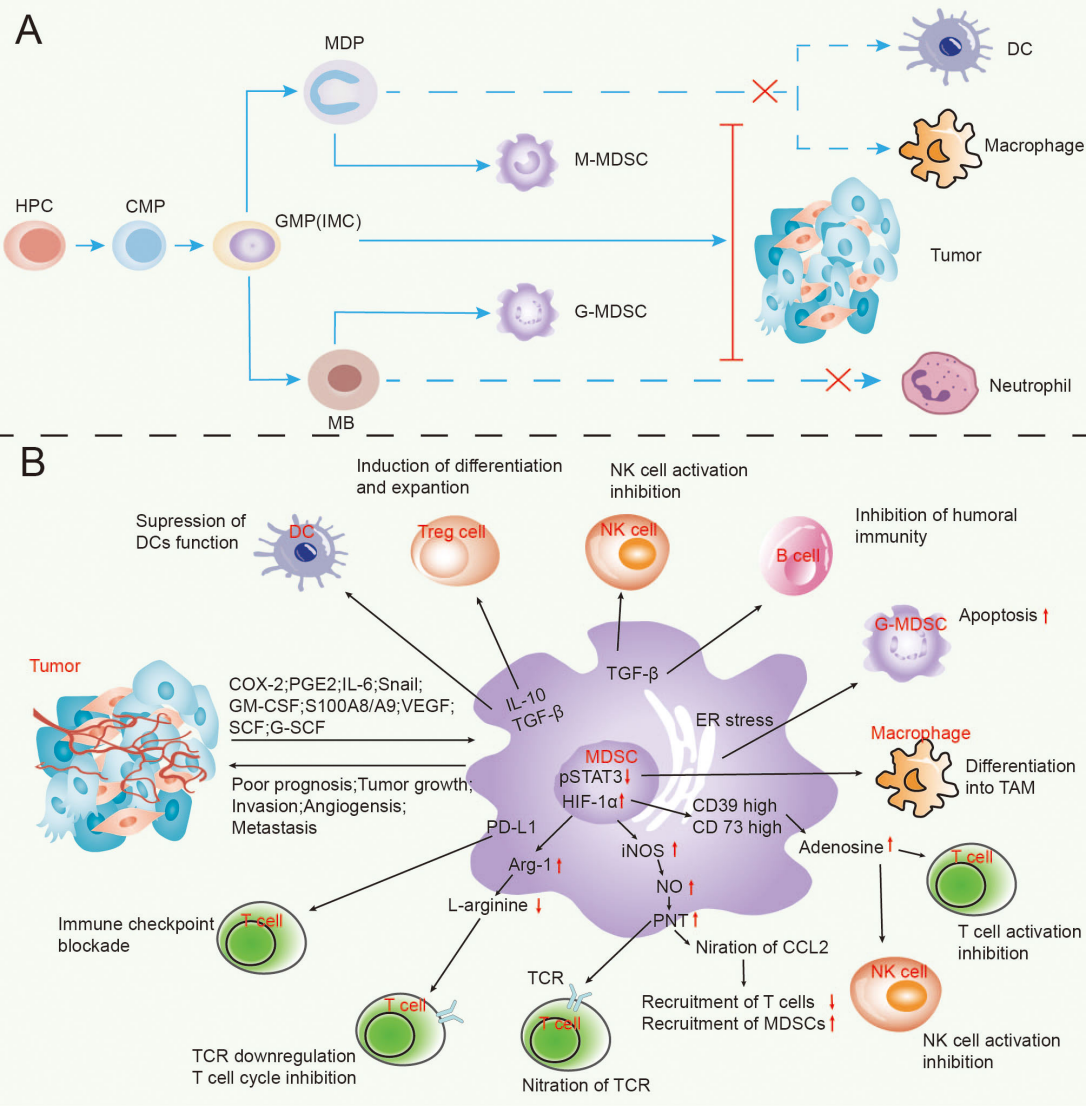
The process of MDSCs’ formation in lung cancer **(A)** and the appliance of MDSCs exert tumour immunosuppressive effects **(B)**.

The other mechanisms for MDSCs’ reduced immunity are explained in findings demonstrating that the regulatory T cells (Tregs) impact tumour immunosuppression. Tregs are considered to be immunosuppressive cells that promote cancer growth, and they are significantly increased in the peripheral blood of NSCLC patients (28). Although there are no proofs to relate MDSCs with the induction of Tregs, they produce a series of cytokines that allow the differentiation of Tregs (29, 30). MDSCs promote the amplification of natural Tregs through the production of IL-10, TGFβ, IFNγ and CD40-CD40L interactions and drive the development of the induced Tregs (24). Sinha et al. have reported that downregulation of the macrophage IL-12 and the IL-10 production is the outcome of T cell polarization to a type 2 pro-tumour phenotype (31). According to sources, the immune cells, specifically the B cells, NK cells, macrophages (Mø) and Treg cells, interact and can be regulated by MDSCs (13). The literature shows evidence for the interaction between DCs, tumour-associated macrophages (TAMS) and MDSCs in the TME. The outcome of this interaction is the enhancement of each cell population’s immunosuppressive activity (24).Thus, in addition to T cell inhibition, MDSCs interfere with the innate immune response by affecting various cells like the NKT, NK, and macrophages (**Figure 1**). Factually, the MDSCs’ impact on NK cells is intricate. One part can inhibit the death of the NK cell by hindering the production of IFN-γ. Importantly, due to the interaction of RAE-1 with NKG2D that takes place on the NK cells’ surface, the other part *via* the expression of RAE-1 can result in NK cells’ activation and provoke their death (24, 32). However, MDSCs’ profile is multi-dimensional. Indeed, they promote tumour cell invasion, drug resistance, tumour angiogenesis, pre-metastatic niche formation, and tumour metastasis while participating in tumour immunosuppressive response (33, 34) .It has been suggested by Yang et al. that the MDSCs’ non-immunosuppressive effects occur mainly *via* the promotion of tumour angiogenesis (35). Epithelial-mesenchymal transition (EMT) has been shown to play a key role in the process of tumor initiation and even metastasis, which can enhance the ability of cancer cells to enter the circulatory system to promote the metastasis of tumor cells. In the process of EMT, tumor cells lose polarity and cell-cell connection, and then enter a state of low proliferation, migration and invasion are enhanced (36). Studies have shown that EMT is related to the number of MDSCs, and EMT transcription factors can attract immunosuppressive cells MDSCs, leading to tumor immunosuppressive microenvironment. In turn, immunosuppressive factors induce EMT in tumor cells (37).The main mechanism is due to the fact that MDSCs and TAM further enhance chronic inflammation in the inflammatory microenvironment, which leads to EMT and enrichment of cancer stem cell-like cells (CSCs) and immune suppression may be the cause of drug resistance and metastasis (38), It is this feedback loop between EMT and immune suppression that promotes tumor progression. Therefore, the combination of immunotherapies targeting immunosuppressive cells such as MDSCs may be a promising treatment for EMT.

### 2.3 Clinical studies confirming the roles of MDSCs in lung cancer

MDSCs represent the most significant immunosuppressive cellular population in individuals with lung tumours. In the stroma of the tumour, these cells limit the healing efficacy of anti-cancer curing approaches through their metabolic pathways and other modalities in response to complex TME (39).There are many clinical studies on MDSCs in lung tumours, and therapeutic strategies targeting MDSCs are also gradually emerging. We have summarized these clinical studies and presented them in **Table 1**.

**Table 1:** Lung cancer clinical studies of MDSCs.

MDSCs	Research population	No. of people	Biological specimens	Results	Reference
M-MDSCs	Advanced NSCLC individuals Healthy people	40 NSCLC patients 20 Healthy People	Peripheral blood	Peripheral blood with stage IV accumulates more than in people with stage III; Individuals with higher accumulation of M-MDSCs have lower survival rates	(40)
G- MDSCs	Individuals with NSCLC Healthy people	90 NSCLC patients 25 Healthy People	Peripheral blood	Patients with low G-MDSCs have better OS	(41)
CD14(+)HLA-DR(-/low)	Individuals with NSCLC	89	Peripheral blood	CD14(+) HLA-DR(-/low) is a novel MDSCs-mediated tumor immunosuppression in NSCLC	(42)
MDSCs	Individuals with SCLC	41	Peripheral blood	Reduction of MDSCs improves the immune reaction to the injection and can enhance the effectiveness of immune interventions against cancer	(43)
M-MDSCs	Individuals with NSCLC	22	Peripheral blood	An increase in M-MDSCs is strongly linked with primary opposition to immunotherapy	(44)
M-MDSCs/G- MDSCs	Individuals with NSCLC Individuals with SCLC Healthy people	26NSCLC patients 16 SCLC patients 8 Healthy People	Peripheral blood	The incidence of M-MDSCs is expressively developed in NSCLC individuals than in SCLC and healthy populations	(45)
CD11b +/CD14^−^/CD15 +/CD33 + MDSC	Individuals with NSCLC Healthy people	173 NSCLC patients 42 Healthy People	Peripheral blood	CD11b +/CD14-/CD15 +/CD33 + MDSCs express their crucial participation in facilitating immunosuppression in NSCLC	(46)
CD14(+)S100A9(+)	Patients with NSCLC	101	Peripheral blood	CD14(+)S100A9(+) is a unique subpopulation of MDSCs that inhibits T cells by arginase, iNOS and the IL-13/IL-4Rα axis	(47)
G- MDSCs	Individuals with NSCLC Healthy people	185 NSCLC patients 20 Healthy People	Peripheral blood	G-MDSCs block T cell proliferation *in vitro*	(48)
CD14(+)HLA-DR(-/low)	Individuals with SCLC Healthy people	42 SCLC patients 37 Healthy People	Peripheral blood	Necessary escalation in the number and incidence of CD14(+) HLA-DR(-/low) MDSCs in the peripheral blood of SCLC individuals, respectively, whose frequency can be considered as a forecaster of meagre prediction in SCLC	(49)
M-MDSCs/G- MDSCs	Individuals with NSCLC	42	Peripheral blood/tumour tissue	Higher frequency of M-MDSCs in tumour tissues compared to normal subjects An increase of M-MDSCs and G-MDSCs in tumours than in peripheral blood Levels of MDSCs in peripheral blood predict recurrence after surgery	(50)
MDSCs	Individuals with NSCLC	46	Peripheral blood	Significantly lower levels of MDSCs after chemotherapy with Bevacizumab	(51)
M-MDSCs	Individuals with NSCLC	176	Peripheral blood	Galactoglucose-9/Tim-3 pathway and mMDSCs for NSCLC are critical to anti-PD-1 primary or secondary resistance	(52)
G- MDSCs	Individuals with advanced NSCLC treated with Nivolumab	53	Peripheral blood	G-MDSCs play as immune biomarkers in NSCLC after the second-line treatment, such as with Nabumab	(53)
MDSCs	Individuals with NSCLC Healthy people	105 NSCLC patients 20 Healthy People	Peripheral blood	Augmented amounts of MDSCs relate to decreased vitality	(54)

Some data show a significant accumulation of M-MDSCs in NSCLC patients, especially in stage IV patients, compared to stage III, with the expected lower survival rate of patients with a higher accumulation of M-MDSCs (40).Other authors found that individuals with NSCLC exhibited an advanced proportion of G-MDSCs, linked with improved survival (41). At the same time, other work has reported on a new human MDSCs subpopulation, CD14 (+) HLA-DR (-/low), in another cohort of individuals with NSCLC. Among those individuals, the incidence and total number of CD14 (+) HLA-DR (-/low) cells in the peripheral blood were considerably augmented compared with those in healthy NSCLC patients (42). The authors hypothesized that this was related to tumour metastasis, adverse reactions to chemotherapy, and tumour immunosuppression. Other work reported the results of different clinical trials with patients with small cell lung cancer (SCLC), in which the depletion of MDSCs improved the immune response to vaccination and further enhanced the effect of immune interventions on cancer (43). It was shown that M-MDSCs from NSCLC patients demonstrated a worse prognosis (44).Seyed Sajjad Zadian et al. demonstrated that the frequency of M-MDSCs in NSCLC individuals was significantly more advanced than that in SCLC and healthy people, which impacted the differential diagnosis of NSCLC (45). Chien-Ying Liu et al. provided evidence of increased CD11b +/CD14 ϫ/CD15 +/CD33 + MDSC in the peripheral blood of NSCLC individuals, which played an essential role in mediating immunosuppression of NSCLC (46).Other authors recognized a unique subgroup of MDSCs in NSCLC patients, namely CD14 (+) S100A9 (+), which inhibited T cells through arginase, iNOS, and IL-13/IL-4Rα axis and were closely associated with adverse effects of chemotherapy (47). Some of the reported studies in **Table 1** measured the amount of MDSCs in the peripheral blood mononuclear cells (PBMCS in untreated NSCLC individuals and made a comparison with the healthy ones (48). Their studies proved that the number of G-MDSC increased in NSCLC individuals, whereas further studies showed that G-MDSCs blocked T cell proliferation *in vitro*. The report by Tian Tian et al. who applied FACS sorting to analyze the peripheral blood of SCLC patients proved that the amount of CD14 (+) HLA-DR (-/low) MDSCs in the peripheral blood of SCLC individuals were considerably augmented. The authors further provided a clue that the amount of these MDSCs were predictors of poor prognosis of SCLC (49).

Interestingly, other authors analyzed tumour resections from NSCLC patients. The results showed that the occurrence of M-MDSCs in individuals with lung tumours was higher and that the accumulation of M-MDSCs and G-MDScs in the tumour site was higher than in peripheral blood. The authors further showed that the MDSCs amount in the peripheral blood could predict recurrence after surgery (50).The effect of the first-line treatment on peripheral blood MDSCs in NSCLC patients was analyzed by other authors. The obtained data presented that chemotherapy with Bevacizumab significantly decreased the levels of MDSCs in the peripheral blood of NSCLC individuals (51). Other authors proposed that the novel targets for immunotherapeutic drug combinations and the treatment of NSCLC through the galectin-9/TIM-3 pathway and mMDSCs were key to anti-PD-1 primary or secondary resistance (52). In patients with Nivolumab treatment, the reported data in another study showed that G-MDSCs could be used as potential immune biomarkers in NSCLC treated with Nivolumab and other second-line therapies (53). In another study by Pauline L de Goeje et al., it was demonstrated that immunoglobulin-like transcript 3 (ILT3) was expressed on MDSCs in fresh peripheral blood mononuclear cells (PBMCS) from individuals with NSCLC. The authors demonstrated that ILT3 cooperated with ligands on the T cells to inhibit T cells, thus augmenting the amount of MDSCs and decreasing survival (54).

Through the above clinical studies, it is not difficult to find that MDSCs are closely related to the prognosis of patients with lung cancer. Compared with normal people, patients with cancer have higher levels of MDSCs, and most of them achieve immunosuppression by inhibiting T cells. When the level of MDSCs in patients is higher, the OS of patients is lower. Lourdes Barrera et al. showed that the OS of patients with low G-MDSCs is better than that of patients with high G-MDSCs through a series of data studies, and the level of G-MDSCs is a potential prognosis of NSCLC disease progression (41). More MDSCs are found in patients with advanced disease, which is associated with poor prognosis. At the same time, primary drug resistance occurs during immunotherapy due to the presence of MDSCs. There are not many studies on the clinical relevance of MDSCs in human cancer, which mainly focus on the correlation between high levels of MDSCS and shorter OS or PFS in different cancers.

Indeed, it is interesting to note that the incidence of M-MDSC and G-MDSC is increased not only in the peripheral blood of patients but also in the neoplastic lesions. Both tumor-infiltrating MDSCs subsets were significantly elevated compared with circulating subsets, confirming that the tumor site had the strongest immunosuppressive effect. In particular, the frequency of tumor infiltration and circulating G-MDSCs correlated with tumor progression (55). In the study by Yoshikane Yamauchi et al., it was noted that the frequency of MDSCs in tumors was higher than that in peripheral blood of the same patients, and this accumulation was associated with increased concentrations of inflammatory mediators involved in MDSC migration to the tumor microenvironment and activation. Moreover, tumor G-MDSCs showed higher expression level of programmed death ligand 1 than the same cells in peripheral blood (50). Xinyu Tian et al. isolated MDSCs from tumor tissues of lung cancer patients by FCM and showed that RUNXOR was significantly associated with MDSCs-induced immunosuppression in lung cancer patients and may be a target for anti-tumor therapy (56). It has been suggested that in a mouse model, monocytic MDSCs can further mature into Tams in the tumor microenvironment, thereby allowing Tams to induce chemotherapy resistance through various mechanisms. Tumor-infiltrating CD68 Tams were analyzed and compared with blood S100A9 MDSCs from the same patients. Indicating their origin from S100A9 MDSCs, it was also found that the percentage of blood S100A9 MDSCs was closely correlated with the counts of S100A9 cells and CD68 TAM in tumor tissue, and patients with higher S100A9 and CD68 cell numbers also showed worse PFS (57).

In addition to clinical studies, several animal trials of MDSCs on lung cancer progression also exist, through which the key role of MDSCs in lung cancer can be supported. In the study of Mi So Park et al., it was found that the main mechanism by which the polypeptide N-acetyl-galactosaminyltransferase (GALNT3) inhibited the development and progression of lung cancer in xenograft and syngeneic mouse models was the ability of MDSCs to infiltrate the tumor site and subsequent angiogenesis, thereby inhibiting the development of lung cancer (58). Although treatment with immune checkpoint inhibitors (ICIs) improves overall survival in a subset of patients with NSCLC, co-occurring KRAS/LKB1 mutations can drive primary resistance to ICIs. Rui Li et al. therefore targeted G-MDSC enrichment as a potential mediator of immunosuppression in LKB1-deficient NSCLC and sensitized tumors to immunotherapy by overcoming MDSCs accumulation with all-trans retinoic acid in a LKB1-deficient NSCLC mouse model (59). Dickson Adah implanted tumors in mice, obtained whole tumors and tumor-derived sorted cells of tumor-bearing mice, and found that malaria infection significantly reduced the proportion of MDSCs and Treg in the lung tumor tissues of treated mice, and inhibited the expansion and activation of MDSCs and Treg in the tumor microenvironment (60). Xiaosan Su et al. also demonstrated that dexmedetomidine (DEX) induced the proliferation of M-MDSCs during the postoperative period in lung cancer patients by inducing spontaneous tumor metastasis in C57BL/6 mice and had a significant pro-angiogenic ability (61). Liran Levy et al. evaluated the effect of splenectomy in several mouse lung cancer models and found that the effect of splenectomy on tumor growth is essentially cell-mediated by MDSCs, which can be used to inhibit the growth of non-small cell lung cancer by depleting MDSCs (62).

Traditional therapeutic approaches for lung tumours are surgery, radio- and chemotherapy. Although some of these treatments are used as first-line therapy, the clinical studies mentioned above have shown that MDSCs can not only exert be immunosuppressive in the TME but could directly promote tumour advancement but also interfere with the prognosis of conventional treatments, thus making it more critical to treat lung cancer by targeting MDSCs. MDSCs were proposed as potential targets for the advance of anti-cancer lung treatment based on the following five aspects (1): promotion of myeloid differentiation (2); blockage of MDSC propagation (3); removal of MDSCs (4); functional decay of MDSCs, and (5) blockade of immune checkpoints (63).Further research on MDSCs showed that miRNAs/lncRNAs could regulate the specialization, propagation, and immunosuppressive roles of MDSCs in TME (64). Therefore, targeting miRNAs and lncRNAs to stop the development and expansion of MDSCs suppressor cells in the tumour environment appears more promising.

In the review’s subsequent chapters, we will focus on lncRNAs and their regulation on the generation, recruitment and immunosuppression of MDSCs.

## 3 Expression and roles of lncRNAs in MDSCs

LncRNAs are a diverse family of non-coding RNAs (ncRNAs). They encompass different ncRNAs like miRNAs, LncRNAs, snRNAs and CircRNAs (65).LncRNAs’ transcripts are longer than 200 nucleotides and are involved in the pathophysiology of many diseases (66). Relevant studies have pointed out that the number of human lncRNAs exceeds the number of protein-coding genes (67). The ENCODE project projected that the human genome contained over 28,000 different lncRNAs, most yet undiscovered (68).Some studies show that lncRNA categories have a high degree of diversity (69), ranging in number from a few hundred to several thousand nucleotides (70).RNA polymerase II transcribes lncRNAs, and according to their genomic localization, mode of action and function can be classified into intronic lncRNAs, intergenic lncRNAs (lincRNAs), enhancer lncRNAs (ELNcRNAs), bidirectional lncRNAs, and sense overlapping lncRNAs (64, 71). Some authors have proven that lncRNAs are mRNA precursors, and compared with mRNAs, ncRNAs show moderate sequence conservation, while lncRNA-pre-mRNA has a significant part in alternative splicing (72, 73). The expression of a variety of lncRNAs is abnormal in various diseases, especially malignant tumours. Some data show that lncRNAs regulate the bone marrow and immune cells. Their regulatory mechanisms are complex and diverse, and they have become vital regulators mediating cell activation, proliferation, differentiation, apoptosis and autophagy (74). Therefore, lncRNAs may have potential diagnostic, prognostic or therapeutic importance. **Table 2** demonstrates the expression of lncRNAs and their regulatory effect on MDSCs.

**Table 2:** Expression of lncRNA and its regulatory effect on MDSCs.

lncRNA	Length	Time of the first report	Mechanism	Impact MDSCs	Related diseases	Reference
PVT1	1.9kb	1985	Hif-1α up-regulates the expression of PVT1 in MDSCs under hypoxia stress	To promote the immunosuppressive effect of MDSCs	Hepatocellular carcinoma, gastric cancer, oesophagal, cervical, bladder, acute myeloid leukopathy	(75)
RUNXOR	260kb	2014	RUNXOR regulates RUNX1 expression by recruiting RUNX1 protein at the 3’ end and binding to promoters and enhancers	Promote the production of MDSCs and immunosuppressive effect	Lung cancer, acute myeloid leukaemia	(56)
lnc-CHOP	1800 bases	2018	Inc-chop binds to CHOP and the C/EBPβ isoform LIP to induce the activity of the C/EBPβ isoform LAP	Promote the production of MDSCs and immunosuppressive effect	Lung cancer, breast cancer, murine melanoma, murine ovarian tumour	(76)
RNCR3	unknown	unknown	RNCR3 binds to Mir-185-5p and releases Chop	Promote the production of MDSCs and immunosuppressive effect	Colorectal cancer, glioma, prostate,	(77)
Olfr29-ps1	963bp	unknown	Olfr29-ps1 promotes the immunosuppressive role and specialization of MDSCs by forming Mir-214-3p after mbA modification	It promotes the immunosuppressive function and differentiation of MDSCs	Lung, breast, pancreatic cancer, urothelial carcinoma	(78)
HOTAIR	2200 bases	2007	HOTAIR induces more CCL2 secretion and promotes the proliferation of MDSCs	It promotes the immunosuppressive function and differentiation of MDSCs	Nasopharyngeal, breast, pancreatic, liver, stomach cancer, non-small cell lung cancer	(79)
HOTAIRM1	1052bp	2009	HOTAIRM1 enhances the expression of HOXA1 in MDSCs	The immunosuppressive function of MDSCs was weakened	Hepatocellular carcinoma, colorectal cancer, Gastric cancer, head and neck neoplasms, Ovarian, Thyroid cancer	(80)
lnc-C/EBPβ	unknown	2018	The binding of LNC-C/EBPβ to C/EBPβ homotypes LIP and WDR5 downregulates IL4il	The immunosuppressive function of MDSCs was weakened	Melanoma, colon cancer, ovarian, breast cancer	(81)
MALAT1	8kb	2003	It acts directly on MDSCs	The immunosuppressive function of MDSCs was weakened	Hepatocellular carcinoma, Endometrial stromal sarcoma, Cervical, Breast cancer, Osteosarcoma, Colorectal cancer	(8)

### 3.1 The lncRNA PVT1 as a potential oncogene in a variety of cancer types

The mouse plasmacytoma variant 1(Pvt1) gene represents a long non-coding RNA located on chromosome 15 (Ch 15) that was reported for the first time in 1985 (82). It is a candidate oncogene coding for a homologous lncRNA to the human Pvt1 gene, localized on Ch 8, specifically near the c-Myc locus on 8Q24. It encodes 52 ncRNA variants, including 26 linear and 26 circular isoforms and six microRNAs and is long 1.9 KB (83, 84). LncRNA PVT1 is recognized as an oncogene in many tumours. Its overexpression is associated with hepatocellular carcinoma, gastric, oesophagal, cervical, and bladder cancer and acute myeloid leukaemia (85–87), Yu Zheng et al. proves that Pvt1 is highly expressed in tumour-expanding G-MDSCs. The results show that lncRNA Pvt1 downregulation considerably blocked the immunosuppressive function of G-MDSC *in vitro*, reducing tumour development and suppressing anti-tumour immune responses. Since Pvt1 expression is augmented in tumour-infiltrated G-MDSCs more than in splenic G-MDSCs, the hypoxic conditions in TME are considered to trigger such a phenomenon. Therefore exposure of splenic G-MDSCs to hypoxic environments reveals an upregulation of both mRNA and protein levels of HIF-1α in these cells. HIF-1α role in the process is clarified by blocking its upregulation by its specific inhibitor YC-1. The results show restored upregulation of Pvt1 and c-myc in hypoxic environments, thus indicating that HIF-1α augmented Pvt1 expression in G-MDSCs cells under hypoxia (75).

### 3.2 The expression of RUNXOR is closely related to MDSC induced immunosuppression in lung tumors

RUNX1 overlapping RNA (RUNXOR) is a lncRNA transcribed by an upstream promoter and overlapping with RUNX1. It significantly controls bone marrow cells’ growth by targeting RUNT-associated transcription factor 1 (RUNX1) (88). LncRNA RUNXOR is about 260 KB long (89). As less research has been done on RUNXOR, dating back as far as 2014, RUNX1 is located on chromosome 21 and is usually disrupted by chromosomal translocations in haematopoietic malignancies. The most common observed translocation is t (8, 21), which is common in acute myeloid leukaemia (90). RUNXOR can regulate RUNX1 expression by recruiting the RUNX1 protein at its 3’ end, and upon binding to promoters and enhancers, it makes it participate in chromosomal translocations in malignant cells tumours (90).

Furthermore, by binding straight to chromatin, RUNXOR orchestrates the long-range chromosomal inner loops. Xinyu Tian et al. proves that the RUNXOR and RUNX1 in MDSCs from the peripheral blood of lung cancer patients are differentially expressed in the tissues around the lung cancer compared to the normal tissues. The study results show that lncRNA RUNXOR is augmented in the lung cancer blood samples, while RUNX1 activity is reversely connected with immunosuppression in MDSCs. Moreover, the activity of RUNXOR is advanced in MDSCs in the lung tumour samples than in the adjacent tissues. The knockdown of RUNXOR also decreased arg1 activity in MDSCs. This suggest that RUNXOR expression is considerably related to MDSC-induced immunosuppression in lung tumours and may be a good aim for anti-tumour therapeutic approaches (56).

### 3.3 lnc -chop regulated MDSCs specialization into M-MDSCs

Lnc-chop is a novel lncRNA identified in MDSCs. It is positioned in the intronic region of the gene on Ch 11. Relevant data indicate that transcription factor C/EBPβ, C/EBP-homologous protein (CHOP) and phosphorylated STAT3 directly influence MDSCs growth (91).CHOP is coded by Ddit3 and takes part in the diminished production of significant factors associated with MDSCs functions, including ARG-1, PNT (peroxynitrite), and superoxide, thereby inhibiting MDSCs activity (92). In addition, lnc-CHOP is associated with lung, breast cancer, murine melanoma, and murine ovarian tumours. The results from the 2018 Yunhuan Gao’s trial notes that lnc-chop would be expressed in MDSCs mediated by factors involved in inflammation and tumour development, while lnc-chop potentiates MDSCs immunosuppressive activity *in vivo* and *in vitro*. These data indicates that lnc -chop regulated MDSCs specialization into M-MDSCs and that M-MDSCs have a more potent immunosuppressive effect. The mechanism behind this is that lnc-chop interacts with CHOP and the C/EBPβ isoform LIP to activate the C/EBPβ isoform LAP, thus lnc-chop triggers enhancement of H3K4me3 in the promoters of Arg-1, NOS2, NOX2 and COX2, which are implicated in MDSCs role in suppressing the immune system in TEM (76).

### 3.4 RNCR3 has potential immunomodulatory functions

RNCR3, also known as LINC00599, is a lncRNA, which is highly conserved in mammals. In tumours, RNCR3 has oncogenic functions and promotes the progression of colorectal, prostate and brain cancers (93).RNCR3 is a crucial regulator of cell propagation, specialization, cell death, metastasis and atherosclerosis (94). Knockdown of RNCR3 leads to increased plasma amounts of TNF-α, CCL2 and IL-6, signifying its potential immunomodulatory functions. Wencong Shang’s trial confirms that RNCR3by acted as a competing endogenous RNA (ceRNA) during MDSC differentiation. Tying to miR-185-5p and releasing Chop stimulated their specialization and immunosuppressive activity. There is a close relationship between RNCR3, miR-185-5p and Chop, and the relationship between the three and how they regulated MDSCs is further elaborated in their subsequent experiments. As mentioned earlier, Chop triggers MDSCs’ specialization and activity *in vivo*, whereas its reduced activity blocks the activity of Arg-1 and iNOS. Therefore, the authors conclude that RNCR3 triggers MDSCs’ specialization by interacting with spongy miR-185-5p to free its target gene Chop. In addition, tumour microenvironmental molecules such as IL-6 triggered RNCR3 expression during MDSCs specialization and further promote their immunosuppressive activity (77).

### 3.5 Olfr29-ps1 can promote the immunosuppressive effect of MDSCs

Olfr29-ps1, a lncRNA distributes in the cytoplasm and nucleus, is a pseudogene, 963 bp in length, located on mouse Ch 4. Its sequence is preserved in vertebrates and is significantly overexpressed in peripheral blood mononuclear cells from individuals with colon and rectal cancer. It is linked with lung, breast, pancreatic, and uroepithelial cancer. Olfr29-ps1 is regulated by the pro-inflammatory cytokine IL6 and tumour-associated factors. IL6 up-regulates Olfr29-ps1 expression in MDSCs, while Olfr29-ps1 is considerably reduced in MDSCs in B16 tumour mice after IL6 knockdown. The Olfr29-ps1 knockdown results in lower NO, H_2_O_2_ and ROS in the cells, whereas increased NO, H_2_O_2_ and ROS are detected in MDSCs that overexpressed Olfr29-ps. Moreover, it is further confirmed that Olfr29-ps1 silencing diminished the protein levels of Arg-1, iNOS, Cox2 and Nox2, while the protein levels of Arg-1, iNOS, Cox2 and Nox2 are augmented in MDSCs that overexpressed Olfr29-ps1. These results signify that Olfr29-ps1 promoted Arg-1 production in M-MDSCs, and H_2_O_2_ in G-MDSCs, contributing to the MDSCs specialization and suppression of the immune system. MDSC overexpressing Olfr29-ps1 in murine models of melanoma resulted in larger, faster and heavier tumour growth. The mechanism by which Olfr29-ps1 affect MDSCs was further investigated. LncRNA pseudogene Olfr29-ps1 can directly sponge mir-214-3p and promote the differentiation and immunosuppressive function of M-MDSC *in vitro* and *in vivo*, which may be achieved by targeting MyD88. Data show that miR-214-3p diminished MyD88 mRNA and protein levels. Furthermore, the interaction between Olfr29-ps1 and miR-214-3p is reliant on the modification of m6A by Olfr29-ps1. Data show that lncRNA Olfr29-ps1 have seven conserved GGAC sequences, the most shared m6A sequences. Therefore, RIP-PCR Olfr29-ps1 is modified by m6A in IL6-induced MDSC. These results confirm that Olfr29-ps1 promoted MDSCs’ functions by forming miR-214-3p after mbA modification (78).

### 3.6 HOTAIR activity was significantly enhanced in lung cancer patients

HOTAIR was discovered in 2007 by Rinn et al. (95). It is a 2.2-kilobase ncRNA located at the HOXC site, specifically in the intergenic place between HOXC11 and HOXC12 genes in the HOXC cluster on Ch 12. HOTAIR recruits a transcriptional corepressor polycombsin Complex 2 (PRC2) to repress the HOXD (homeobox gene cluster D) expression (96). In individuals diagnosed with lung tumours, HOTAIR activity is considerably augmented. Therefore, it is considered a new controller of lung tumours, which has great significance in the possible therapeutic approaches to lung cancer (97). HOTAIR is also overexpressed in nasopharyngeal carcinoma, breast, pancreatic, liver, gastric and non-small cell lung cancer (98).HOTAIR was proven to be linked with MDSCs functions in hepatocellular carcinoma lines. In HCC patients, MDSCs exerted their immunosuppressive functions by inducing regulatory T cells. Other authors demonstrated that HOTAIR induced the secretion of CCL2, which recruited TAM and MDSCs, in PBMCs co-cultured with HOTAIR overexpressing cells. It was also shown that HOTAIR played a crucial role in promoting macrophages and MDSCs by secreting cytokines and chemokines from HCC cells (79).

### 3.7 HOTAIRM1 is highly active in different tumors

HOTAIRM1 (HOXA transcribed antisense RNA bone marrow specific 1) was discovered by (Xueqing Zhang et al., 2009). It is 1052bp in length and is located in the HOXA gene cluster between HOXA1 and HOXA2 on human Ch 7 (99). Initially, it was considered the most prominent intergenic transcript of granulocyte differentiation and up-regulation in NB4 promyelocytic leukaemia. Other authors proved that it was overexpressed in specific myeloid lines (100). Recent data show that HOTAIRM1 is a lncRNA that is abnormally active in different tumours and is related to hepatocellular carcinoma, colorectal, gastric, head and neck, ovarian, thyroid cancers, etc (101).Some authors detected that HOTAIRM1 expression was significantly reduced in tumour tissues. In addition, overexpression of HOTAIRM1 downregulated Arg1 expression levels and inhibited the effect of MDSCs. However, when HOTAIRM1 was overexpressed, the HOXA1, a target gene of HOTAIRM1, was significantly enhanced, suggesting that HOTAIRM1 could trigger HOXA1 expression in MDSCs. In contrast, its silencing or HOXA1 expression in MDSCs significantly reduced the frequency of MDSCs and their inhibitory function. It was further found that HOXA1 overexpression downregulated the activity of the immunosuppressive molecule Arg1 and ROS production in MDSCs and that HOXA1 overexpression enhanced CD4+ Th1 and CD8+ CTL cell initiation so that HOXA1 overexpression enhanced the anti-tumour T-cell response and suppressed the immunosuppressive effect of MDSCs, thereby delaying tumour progression (80).

### 3.8 Lnc C/EBPβ was significantly increased in G-MDSC

Lnc-c/EBPβ is located on Ch 1 and 4. It is represented by three subtypes of C/EBPβ: liver-rich activator protein (LAP) and liver-rich repressor protein (LIP) (102). Studies on LNC-C/EBPβ are scarce. In 2018 some authors reported on the isolation of MDSCs from mice carrying melanoma, colon, ovarian and breast cancer. MDSCs subsets were classified to analyze the expression of RNC-C/EBPβ in different MDSC subsets. The results demonstrated that lnc-C/EBPβ was found in G-MDSC, M-MDSC and macrophages, and the amount of lnc-C/EBPβ was significantly augmented in G-MDSCs. It has been suggested that ARG-1, NOS2, NOX2 and COX2 activity was controlled by C/EBPβ, which lessened MDSCs’ immunosuppressive functions (103). The underlying mechanism included LNC-C/EBPβ knockdown, which changed the transcriptional activity of several genes, like interleukin4-induced gene-1 (IL4i1). However, LNC-C/EBPβ binding to C/EBPβ homotypes LIP and WDR5 was necessary for LNC-C/EBPβ-mediated IL4il silencing. The results showed that the expression of LNC-C/EBPβ was controlled by IL 6, while LNC-C/EBPβ potentially promoted the differentiation of PMN-MDSC. Furthermore, LNC-C/EBPβ hindered the specialization of MDSCs into M-MDSCs (81).

### 3.9 MALAT1 regulates the differentiation of MDSCs

MALAT1 (metastasis-associated lung adenocarcinoma transcript 1) is an extensively investigated lncRNA, especially in tumour biology. It is also recognized as NEAT2 (a nuclear-rich transcript 2), mainly localized in the nucleus and highly preserved among animals. In humans, it is found on chromosome 11q13. Its main transcript length is about 8kb in humans and 6.7kb in mice (104). It was one of the earliest lncRNA genes discovered, and some data linked it with metastasis in NSCLC individuals. It was overexpressed in NSCLC patients and appeared predictive of early-stage NSCLC in individuals with a high risk of metastasis. However, MALAT1 was not only highly expressed in lung cancer but participated in the progression and expansion of hepatocellular carcinoma, endometrial stromal sarcoma, cervical, breast cancer, osteosarcoma, colorectal cancer and others (105). Furthermore, MALAT1 regulated the molecular signalling pathways that drove cell division, apoptosis, cell cycle, metastasis, invasion, and immune response. It was linked to tumour site, size, salinization and cancer stage, so the MALAT1 abnormal expression in tumour tissues or body fluids could be used for diagnostic and prognostic purposes (106).Other authors proved that the average transcription activity of MALAT1 in PBMCs isolated from individuals with lung tumours was considerably reduced and was adversely linked with the amount MDSCs. These results proved that MDSCs and CTL were negatively correlated in PBMCs of individuals with lung tumours. However, according to Qinfeng Zhou et al., MALAT1 levels were not directly correlated with MDSCs and CTLs in PBMCs of patients with lung tumours. Therefore, it could be concluded that MALAT1 regulated the differentiation of MDSCs. This study also demonstrated that MALAT1 knockdown increased the number of MDSCs by regulating their differentiation, which provided a new understanding of the development of lung tumours and proposed a possible target for supplementary diagnosis and management of lung tumours (8).

Tumor microenvironment, such as hypoxia, the presence of IL-6 and other inflammatory factors, may promote the expression of lncRNA in MDSCs. For example, IL-6 or tumor-related factors can induce the overexpression of lnc C/EBPβ, LNC-CHOP, Olfr29-ps1, Pvt1 and RNCR3 in MDSCs, while chronic and low-dose stimulation of inflammation and tumor factors can also promote the down-regulation of lncRNAs. For example, HOTAIRM1 and MALAT1 are down-regulated in MDSCs from lung cancer patients. These microenvironmental factors produce different effects. From the above introduction, we can know that lnc-CHOP, Olfr29-ps1, Pvt1, RUNXOR, HOTAIR and RNCR3 can promote the immunosuppressive function and differentiation of MDSCs. lnc-C/EBPβ, HTOAIRM1 and MALAT1 blocked the immunosuppressive and differentiation functions of MDSCs, resulting in completely different effects. At the same time, some studies have shown that lncRNAs(such as Olfr29-ps1, lnc-CHOP, RNCR3 and RUNXOR) can participate in the first stage of MDSCs expansion and then participate in the second stage of MDSCS activation due to the identification of different targets, while Pvt1 can only participate in the second stage. In addition, lncrnas can also play cell-to-cell immunosuppressive and tumor-promoting roles through exosomes secreted by MDSCs.

## 4 The roles of lncRNAs in lung cancer

**Figure 2:**
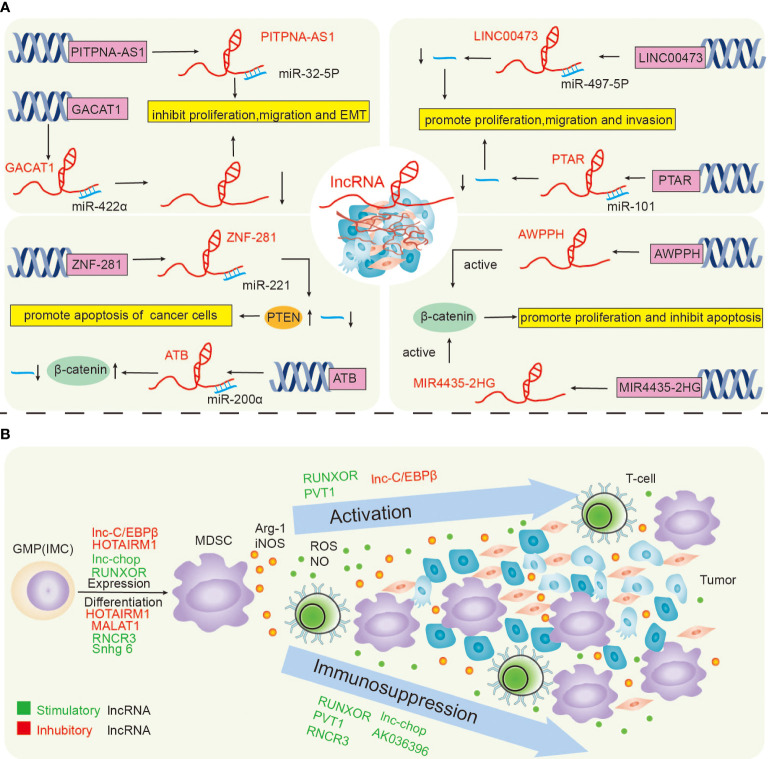
LncRNAs directly regulate lung cancer **(A)**, and the lncRNAs regulate lung cancer by regulating MDSCs **(B)**.

LncRNAs control the immune cells’ functions by various mechanisms. The regulation of MDSCs in various tumour tissues by lncRNAs can promote their application in immunoregulatory anti-cancer therapy or as biomarkers. LncRNAs have dual roles in cancer. They can block or trigger its development. For example, the transmission of lncRNAs in exosomes triggers drug non-responsiveness. Data show that LncRNA H19 encapsulated into exosomes and unambiguously facilitated by hnRNPA2B1 is moved to non-resistant NSCLC cells, resulting in non-responsiveness to Gefitinib (36).In our study, we explored and summarized the direct regulation of lung cancer by lncRNAs and further investigated the indirect regulation of lncRNAs on lung cancer by regulating MDSCs (**Figure 2**).

### 4.1 LncRNAs directly regulate lung cancer

Taking lncRNAs as an entry point holds the promise of further improving the survival of individuals with lung tumours. Unfortunately, there is a lack of lncRNA-related lung cancer studies. Here, we summarize the available information on lncRNAs’ regulation of lung cancer and provide the findings in **Table 3**.

**Table 3:** Regulation of lncRNAs in lung cancer.

lncRNA	Lung cancer subtypes	Effects on lung cancer	Mechanism	Reference
PITPNA-AS1	NSCLC	Silencing inhibits NSCLC cell proliferation, metastasis, and epithelial-mesenchymal transition	To inhibit NSCLC progression by silencing PitPNA-AS1 by targeting Mir-32-5p	(107)
ZEB2-AS1	NSCLC (A549)	It is significantly expressed in NSCLC tissues and triggers metastasis and epithelial-mesenchymal transition of NSCLC tumour cells	unknown	(108)
NEAT1	NSCLC	Modulates sensitivity to iron death in NSCLC cells	unknown	(109)
ZNF281	NSCLC	Blocks proliferation of cancer cells and triggers cell death	Overexpression of ZNF281 and PTEN can accelerate cell apoptosis and inhibit cancer cell proliferation. ZNF281 can down-regulate Mir-221 in NSCLC to up-regulate PTEN	(110)
LINC00473	NSCLC (A549、 H1299)	It can promote cell propagation and metastasis and inhibit cell death in NSCLC	LINC00473 promotes the progression of NSCLC by regulating the ERK/P38 and MAPK signalling pathways and the expression of Mir-497-5p	(111)
PTAR	NSCLC(A549)	It indorses cell division and metastasis of NSCLC	LncRNA PTAR triggers the growth of NSCLC cells by inactivating Mir-101	(112)
GACAT1	NSCLC	Down-regulation blocks cell division and triggers cell death in NSCLC	The expression of GACAT1 in NSCLC was decreased by sponging Mir-422a to inhibit the progression of NSCLC	(113)
AWPPH	NSCLC	Overexpression triggers cell propagation and blocks cell death in NSCLC	LcRNA AWPPH triggers NSCLCs propagation by stimulating the Wnt/β-catenin signalling pathway	(114)
PCAT 19	NSCLC	Overexpression resulted in increased proliferation of NSCLC cancer cells	Overexpression of PCAT 19 can down-regulate p53 and promote the progression of NSCLC	(115)
LncRNA ATB	NSCLC	Promote apoptosis of NSCLC cancer cells	LncRNA ATB inhibited the activity of Mir-200a and promoted the β-catenin transcription in reverse	(116)
lncRNA MIR4435-2HG	adenocarcinoma of lung	Mir4435-2HG knockdown considerably blocked the propagation and metastasis of lung tumour cells	Mir4435-2hg binds to β-catenin to stop its destruction controlled by the proteasome system, thereby controlling the EMT and cancer stem cell properties in lung tumours	(117)

The roles of lncRNA PITPNA antisense RNA 1 (PITPNA-AS1) were studied in individuals with NSCLC. The results showed that the transcription levels of PitPNA-AS1 in NSCLC tissues were overexpressed in NSCLC tissues. However, its silencing blocked NSCLC propagation and metastasis. An interesting connection between PitPNA-AS1 and microRNA (miR)-32-5p was detected, proving that PITPNA-AS1 downregulation inhibited the progression of NSCLCs by directing Mir-32-5p. This suggested PITPNA-AS1 as a diagnostic and prognostic biomarker of NSCLC (107). Other authors proved that the lncRNA ZEB2-AS1 was overexpressed in NSCLC patients, which stimulated the epithelial-mesenchymal transition (EMT) in these patients, proving that lncRNA ZEB2-AS1 may become a new diagnostic, prognostic and therapeutic factor (108). Hongxia Wu et al. proposed that targeting the lncRNA NEAT1 could be a possible treatment for NSCLC (109). Some studies have pointed out that the zinc finger protein (ZNF) 281 could be a tumour suppressor lncRNA in glioma. Xin Lu et al. followed up on patients for 5 years to analyze the role of ZNF281 in NSCLC. They found that ZNF281 up-regulated the phosphatase and tensin homolog (PTEN) by down-regulating Mir-221 in NSCLC, thus constraining cancer cell propagation and death (110). The lncRNA LINC00473 was overexpressed in lung tumour tissues and NSCLC cells (A549 and H1299), resulting in a low 5-year patients’ OS (overall survival). Studies on lung tumour tissues demonstrated that LINC00473 interacted with Mir-497-5p and blocked its activity, thus promoting the propagation of NSCLC cells (111). To explore the relationship between lncRNA transforming associated RNA (PTAR) and Mir-101 in NSCLC, Wenjun Yu et al. conducted a series of studies during which they proved that lncRNA PTAR was up-regulated in NSCLC cells and could be combined with Mir-101 to inactivate it to stimulate the growth of NSCLC cells (112). GACAT1 (the lncRNA gastric cancer-associated transcript 1) plays a carcinogenic role in different types of cancer. It is overexpressed in NSCLC tissues, which may be associated with the adverse outcome of NSCLC patients. This finding delivers new knowledge for developing novel therapeutic approaches for NSCLC (113). AWPPH is a recently revealed lncRNA, which can be highly expressed in NSCLC tissues, thus stimulating the propagation of NSCLC cells, and considerably inhibiting the survival rate of patients with high AWPPH expression (114).LncRNA prostate cancer-associated transcript (PCAT) 19 also has a certain effect on the progression of NSCLC, PCAT19 was found overexpressed in NSCLC, which increased NSCLC cell proliferation and promoted the progression of NSCLC (115).Ting Wang et al. discovered the role of lncRNA-ATB in NSCLC using the *in vitro* cultured NSCLC NCI-H838 cell line. Their findings revealed that lncRNA-ATB promoted lung cancer progression by inhibiting miR-200a expression and reversed the promotion of β-linked protein expression to promote apoptosis in NSCLC cells (116).The newly discovered lncRNA SET binding factor 2 antisense RNA 1 (LncRNA SBF2-AS1) is involved in the progression of many cancers like lung, breast, hepatocellular carcinoma, thyroid, gastric, colorectal cancers and others (118). Interestingly, Weilong Ye et al. identified 13 lncRNAs related to Gefitinib metabolism and applied them to build the prognostic model of NSCLC patients (119). This proves that lncRNA studies are not limited to particular tumour tissue, and more and more studies have proposed new ideas for the diagnosis, treatment and prognosis of lncRNAs.

Ghada Mohamed Gamal El-din et al. verified the expression of serum markers RAB27A mRNA and RNA-RP11-510m2 in 20 individuals with lung tumours, 10 individuals with COPD and 10 controls in good physical shape. The results showed that the serum exosome RAB27A mRNA was positively correlated with lung cancer, whereas NRC-RNA-RP11-510m2 was negatively correlated, which could be developed as biomarkers for diagnosis and prognosis of lung tumours (120).LncRNAs HOTAIR has been shown to induce tumorigenesis of several cancer types. Chunlin Ke et al. explored the relationship between the four types of lncRNA HOTAIR and the susceptibility to lung cancer. One thousand seven hundred fifteen individuals with lung tumours and 2745 healthy subjects were recruited. The results proved that HOTAIR was important in lung cancer screening and prognosis prediction, especially for people with high-risk factors (121). Sik1-lnc is another lncRNA adjacent to salt-inducible kinase 1 (SIK1) and can be abnormally expressed in lung cancer. Other authors (Liu Yang et al.) studied the expression of SIK1 and SIK1-LNC in samples from lung cancer patients and established that the transcription activity of SIK1 and SIK1-LNC was decreased.

Moreover, SIK1-LNC considerably repressed the propagation of lung cancer cells, signifying that SIK1-LNC served as a novel biomarker and target for lung cancer therapeutic approaches (122).TSLC1, as a tumour suppressor gene in various cancers, is considerably repressed in NSCLC tissues and cell lines. The increased expression of TSLC1 inhibited the viability, migration and invasion of NSCLC cells. Other authors explored the biological mechanism of the antisense RNA of TSLC1, namely lncRNA RP11-713B9.1, in the development and progression of NSCLC. They analyzed the tumour tissues of 46 NSCLC patients. They established that the activity of RP11-713B9.1 was undoubtedly connected with TSLC1 (a tumour suppressor gene), while the overexpression of RP11-713B9.1 led to a substantial overexpression of TSLC1. In other words, the inhibition of RNA RP11-713B9.1 transcription backed NSCLC cell survival (123).

### 4.2 LncRNAs regulate lung cancer by modulating the functions of MDSCs

MDSCs are indispensable for the occurrence and progression of lung tumors. Therefore it is very logical to assume that lncRNAs could regulate lung tumors indirectly b modulating MDSCs’ activity. In a study by Qinfeng Zhou et al., the levels of MDSCs and ARG-1 were significantly increased in PBMCs of lung cancer patients. Simultaneously, the relative expression of lncRNA MALAT1 PBMCs of individuals with lung tumors was considerably reduced. The direct effect of MALAT1 on MDSCs was further confirmed by siRNA interference of MALAT1 expression, which resulted in the inhibition of MDSCs expansion (8). RUNXOR lncRNA is significantly related to MDSCS-induced immunosuppression in individuals with lung tumors and could serve as therapeutic targets. In another study, the expression of lncRNA RUNXOR in PBMCs isolated from individuals with lung tumors was studied by qRT-PCR. The level of RUNXOR in individuals with lung tumors was found to be augmented. Moreover, the authors detected a differential expression of RUNXOR in squamous cell lung and lung adenocarcinoma, thus suggesting that RUNXOR could be used to differentiate lung tumor types. Furthermore, the analysis of the peripheral blood of individuals with lung tumors showed that RUNXOR transcription was augmented with the increase of MDSCs percentage and Arg1 levels but was decreased with the increase of the Th1/CTL cell ratio. This suggested that the transcriptional activity of this lncRNA controlled the immunosuppressive function of MDSCs in these individuals (56). In another study, it was shown that lncRNA Snhg6 mainly exists in the cytoplasm.Furthermore, they proved that Snhg6 controlled MDSCs specialization by decreasing the stability of EZH2 without affecting their immunosuppressive functions (124). As mentioned above, the up-regulation of the lncRNA HOTAIRM1 down-regulated the inhibitory molecules in MDSCs. Other authors proved that HOTAIRM1 was conveyed in different types of lung cancer, especially in lung adenocarcinoma, and its transcription activity was considerably diminished in MDSCs from tumor tissues. Moreover, they showed that when HOTAIRM1 was overexpressed, Arg1 expression levels in MDSCs were down-regulated, thus inhibiting the propagation of MDSCs in lung tumors (125). In another study, the authors established that lncRNA F730016J06Rik (AK036396) was overexpressed in G-MDSCs, whereas its knockdown promoted the maturation of G-MDSCs and reduced their immunosuppression functions. Data show that the Fcnb protein can bind to some proteases to promote the production of ROS and Arg1 by MDSCs through the lectin pathway in granulocyte. The last accelerates the migration of MDSCs to tumor sites. Therefore, AK036396 can enhance the stability of Fcnb protein through the ubiquitin-proteasome system. Thus, the maturation and function of G-MDSCs can be regulated to accelerate immune suppression (126). Yu Zheng et al. implanted Lewis lung cancer (LLC) cells into mice, isolated MDSCs by microbeads and flow cytometry, and measured the transcriptional activity of Pvt1. The authors detected that Pvt1 was overexpressed in G-MDSCs derived from cancer samples. The immunosuppressive effect of tumor-infiltrating MDSCs was mainly due to increased Arg1 transcription and production of nitric oxide by HIF-1α. Coincidentally, Yu Zheng et al. first identified Pvt1 as a HIF-1α target in G-MDSCs of LLC mice cells under hypoxia. RNA interference of Arg1 diminished its activity, ROS production in G-MDSCs decreased, and the antitumor T-cell response was restored. From this perspective, the authors concluded that targeting Pvt1 attenuated G-MDSCS-mediated immune suppression (75). LncRNAs regulate tumors far beyond lung cancer. The proof comes from Zohreh Khodaii et al., who explored the LncRNA-Mir-mRNA complex to find new targets in a rectal tumor. The depletion of Lactobacillus acidophilus in individuals with rectal tumors induced the transcription of the lncRNA-Mir-mRNA network, which delivers new observing and treatment methods for rectal cancer patients (127). Other authors performed biopsies of tumor and non-tumor tissues from patients with gastric cancer. They showed that lncRNA PVT1 expression was increased, whereas the lncRNA ZFAS1 expression was decreased compared with non-tumor parts. PVT1 and ZFAS1 were biomarkers for detecting and treating gastric cancer cases (128). Furthermore, the lncRNA MEG3 was reported as a tumor suppressor in breast cancer. Battseren Bayarmaa et al. evaluated the impact of MEG3 polymorphism on neoadjuvant therapy in144 patients with breast cancer. The results showed that MEG3 polymorphism was associated with the chemotherapy response and toxicity of paclitaxel and cisplatin. This indicated that MEG3 polymorphism has the potential as a prognostic marker for breast cancer individuals (129). Xianmin Guan et al. obtained bone marrow samples from 146 pediatric patients with acute myeloid leukaemia (AML) and 73 patients with non-hematologic malignancies. They measured lncRNA-SOX6-1 expression to examine the association between lncRNA-SOX6-1 and AML. SOX6-1 transcription was augmented in the AML patients compared to the healthy volunteers, which promoted cell propagation while inhibiting cell death and was related to worse risk diversification and poorer treatment outcomes (130). Similarly, Zhenqing Tan et al. studied the relationship between INK4 expression and the clinical characteristics and prediction of AML in patients, they studied the transcription of ANRIL in bone marrow mononuclear cells (BMMCs) in 178 patients with initial AML and 30 healthy donors. Compared with healthy people, lncRNA ANRIL levels were increased in AML patients, and those with augmented ANRIL transcription had smaller event-free survival (EFS) and OS. Therefore, ANRIL was proposed as a biomarker for AML. Moreover, it has clinical relevance in assisting the diagnosis, treatment and prognosis prediction of AML and identifying potential drug targets of AML (131).

## 5 Summary and outlook

The development mechanisms of tumour cells are diverse, and the tumour microenvironment changes are also complex and miscellaneous. To successfully implement tumour immunotherapy, tumour suppressors must be removed. Recent data have shown that MDSCs are the chief controllers of cancer immune responses and inflammation in individuals with tumours as they intensely constrain the antitumor immune response of CD4+ T cells, CD8+ T cells and NK cells, thus triggering tumour growth. MDSCs play critical roles not only in lung cancer carcinogenesis but also in its progression and prognosis. Therefore, MDSCs are an attractive therapeutic target because they are carefully related to adverse effects. With the discovery of many novel lncRNAs and the general studies on their roles in different pathologies, particularly cancer, lncRNA research has become a new trend. Factors generated by the tumour and its accompanying hypoxia or inflammatory TME may sustain the expression of some non-coding RNAs. Based on lncRNAs, treatment strategies targeting MDSCs can recover our understanding of MDSCs and disclose new tumour propagation instruments.

Furthermore, the study of novel lncRNAs that regulate the activity of MDSCs is expected to enable their application in immunoregulatory therapy or as biomarkers. In particular, recent data proved that lncRNAs play a significant part in NSCLC development, which will provide a new direction for our subsequent research on lung cancer. However, the complex biological mechanism of MDSCs also poses new challenges for targeted therapy. In addition, the functional connection between lncRNAs and MDSCs does not seem strong enough, the investigations on the way the lncRNAs regulate MDSCs are yet in their infancy, and the clinical research on lncRNA-related lung cancer is very little. Nevertheless, it is hoped that with the development of social science and the progress of medical technology, more clinical studies in this field can be piloted to approve the possibility of regulating MDSCs in lung cancer by lncRNAs. The last will deliver novel ideas for lung cancer treatment and bring more benefits to lung cancer patients.

## Author contributions

XP and JH designed and supervised the study. YL and YH reviewed the references. YL and XP wrote the manuscript. YL, YZ and TL contributed to tables and figures, XP and JH revised the manuscript. XP acquired funding. All authors contributed to the article and approved the submitted version.
